# A Robust Recurrent Neural Networks-Based Surrogate Model for Thermal History and Melt Pool Characteristics in Directed Energy Deposition

**DOI:** 10.3390/ma17174363

**Published:** 2024-09-03

**Authors:** Sung-Heng Wu, Usman Tariq, Ranjit Joy, Muhammad Arif Mahmood, Asad Waqar Malik, Frank Liou

**Affiliations:** 1 Department of Mechanical Engineering, Missouri University of Science and Technology, Rolla, MO 65409, USA; swm54@umsystem.edu (S.-H.W.);; 2 Intelligent Systems Center, Missouri University of Science and Technology, Rolla, MO 65409, USA; 3 National Strategic Planning and Analysis Research Center (NSPARC), Department of Electrical and Computer Engineering, Mississippi State University, Starkville, MS 39759, USA

**Keywords:** directed energy deposition, surrogate model, recurrent neural network, melt pool characterization, thermal history

## Abstract

In directed energy deposition (DED), accurately controlling and predicting melt pool characteristics is essential for ensuring desired material qualities and geometric accuracies. This paper introduces a robust surrogate model based on recurrent neural network (RNN) architectures—Long Short-Term Memory (LSTM), Bidirectional LSTM (Bi-LSTM), and Gated Recurrent Unit (GRU). Leveraging a time series dataset from multi-physics simulations and a three-factor, three-level experimental design, the model accurately predicts melt pool peak temperatures, lengths, widths, and depths under varying conditions. RNN algorithms, particularly Bi-LSTM, demonstrate high predictive accuracy, with an R-square of 0.983 for melt pool peak temperatures. For melt pool geometry, the GRU-based model excels, achieving R-square values above 0.88 and reducing computation time by at least 29%, showcasing its accuracy and efficiency. The RNN-based surrogate model built in this research enhances understanding of melt pool dynamics and supports precise DED system setups.

## 1. Introduction

Directed energy deposition (DED) is an additive manufacturing (AM) technique for metals that creates parts by melting metal feedstocks with concentrated thermal energy [1,2]. Compared to the laser powder bed fusion process, DED is more cost-efficient and capable of producing parts with greater efficiency and adaptability [3]. These remarkable characteristics make DED an attractive option for rapid prototyping, manufacturing functionally graded materials, and repairing high-value components [4]. Specifically, DED excels in repairing worn or damaged components, thereby extending the service life of industrial and aerospace equipment by restoring structural integrity and functionality [5]. Over the last decade, DED’s usage has expanded in the defense, manufacturing, and automotive industries [6]. For instance, DED has been employed to repair airfoils in airplane engines [7]. The DED market size is projected to reach more than USD 700 million by 2025 [8]. Despite DED’s advantages over other AM techniques, challenges remain in minimizing defects during printing. Factors contributing to defect generation include gas entrapment, insufficient melting, and unstable melt pool generation [9,10]. Comprehending the thermal behavior and melt pool generation in relation to process parameters is essential for reducing defects during DED printing [11].

In DED, the melt pool is defined as the regime where metal particles are melted during laser–material interaction, generating an orbicular droplet [2,12]. Within the molten pool, the thermal distribution plays a crucial role in defining the microstructure and defects of the manufactured part [13]. In the case of a small molten pool, a relatively reduced thermal distribution can result in inadequate adjacent melt pools’ overlap, leading to the lack of fusion defects [14]. Additionally, an irregular molten pool caused by elevated energy density can cause keyhole formation, leading to substantial material vaporization [15,16]. The dense plasma plume results in a recoil force on the molten material which leads to gas entrapment, creating defects [17,18]. Attaining and monitoring optimal thermal distribution is essential for an appropriate melting flow within the molten pool [19]. DED often encounters non-uniform thermal distribution along with rapid heating and slow cooling cycles, developing anisotropic microstructures, characterized by porosity and uneven grains [20]. The uneven grains affect the mechanical properties negatively [21,22]. For the DED process, the thermal distribution within the molten pool can be monitored using sensors such as thermocouples, IR cameras, and pyrometers. IR camera, in combination with image processing, was applied to observe thermal distribution within the melt pool. Comparatively reliable results were obtained at a 100 kHz sampling rate as well as 20 µm resolution [23]. In addition, the IR camera and pyrometers can monitor radiation from moving bodies and capture thermal distribution without surface contact, thus assisting in situ monitoring of the DED process [24]. On the other hand, thermocouples are flexible and resource-effective compared to other sensing devices. However, direct contact is required for thermocouples, which limits their usage [25].

To predict molten pool thermal distribution, researchers have explored multi-physics and machine learning-based approaches [26,27]. In multi-physics techniques, FEM and analytical methods have been elaborated. On the one hand, an extensive multi-physics FEM model may provide reliable results on the verge of computational [28]. On the other hand, a simplified FEM model faces limitations owing to incomplete multi-physics involved in simulation analysis [29,30]. Furthermore, the accuracy of the FEM model is also affected by factors such as element type, initial and boundary conditions, and meshing size [29]. In addition, the analytical techniques utilize multi-physics equations solved based on the initial and boundary conditions, simulating the thermal distribution and melt pool formation in the DED process [31]. These methods are unreliable due to mass and volume variation with time and uncertainties involved in DED processes.

Machine learning (ML)-based approaches have demonstrated significant advantages in modeling the intricate thermal distributions and melt pool formations essential to Directed Energy Deposition (DED), achieving solid accuracy and efficiency [32]. These approaches significantly reduce the high costs associated with extensive experimental procedures in research and development and alleviate the burden of lengthy computational times typically required by traditional simulation methods [33]. ML-based models are fundamentally data-driven, analyzing the relationship between each process parameter, like laser power, scanning speed, powder feed rate, and its outputs, such as thermal distribution and mechanical properties [34]. The data for training these models are usually collected from experiments or simulations, and the predictive insights provided by ML models greatly enhance the scalability of applications across various scenarios [35]. Various ML algorithms, such as SVM, clustering, and artificial neural networks, have been utilized to predict melt pool characteristics [36,37]. In addition, the defects of printed parts can be detected by predicting the melt pool dimension [38]. Despite these advancements, the dynamics of melt pools pose complex challenges. Primarily, the acquisition of large, robust datasets necessary for training these models is prohibitively expensive and time-intensive [39]. Additionally, current research inadequately addresses the sequential nature of melt pool dynamics, highlighting a critical need and understanding for more sophisticated applications of recurrent neural network (RNN) algorithms. Furthermore, the computational demand and memory requirements of these models also need optimization to enhance their reliability and robustness.

To address these challenges, this research introduces a pioneering RNN-based surrogate model designed specifically to predict both the thermal history and the geometric characteristics of melt pools in DED. The comprehensive framework that incorporates a factorial design of experiments, multi-physics modeling, refined data processing, and rigorous surrogate model training, evaluation, and comparison are proposed for this research. This innovative approach deepens the understanding of complex melt pool dynamics and significantly advances the operational capabilities of DED systems. It marks a substantial progression in the field, enhancing the precision and efficiency of ML-based surrogate models and facilitating their practical application in optimizing DED processes.

## 2. Methodology

**Figure 1:**
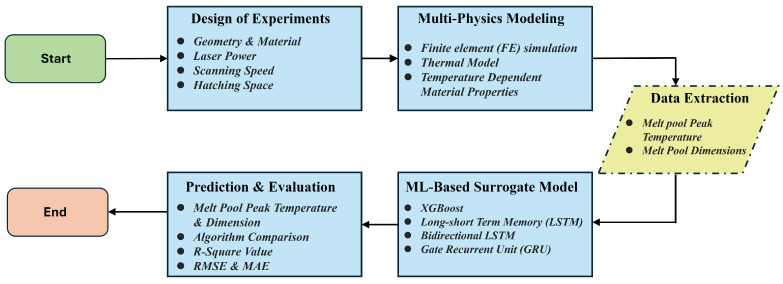
Proposed flow chart of current research.

The method used to develop the robust machine learning-based surrogate model for predicting melt pool thermal history and characteristics is presented in Figure 1. The process begins with the design of experiments, focusing on various parameters such as geometry, material, laser power, scanning speed, and hatch spacing. This is followed by multi-physics modeling, which includes finite element (FE) simulations and thermal modeling with temperature-dependent material properties. Key data points such as melt pool peak temperature and dimensions are extracted for building the surrogate model. In this research, the surrogate model is machine learning-based, employing multiple machine learning algorithms including Extreme Gradient Boosting (XGBoost), Long Short-Term Memory (LSTM), Bidirectional Long Short-Term Memory (Bi-LSTM), and Gated Recurrent Unit (GRU) to ensure accurate predictions of melt pool thermal history and dimensions. The evaluation and comparison of each algorithm are based on R-square values, Root Mean Square Error (RMSE), and Mean Absolute Error (MAE), ensuring robust model performance. A detailed description of each section is provided in the following content.

### 2.1. Design of Experiments

**Figure 2:**
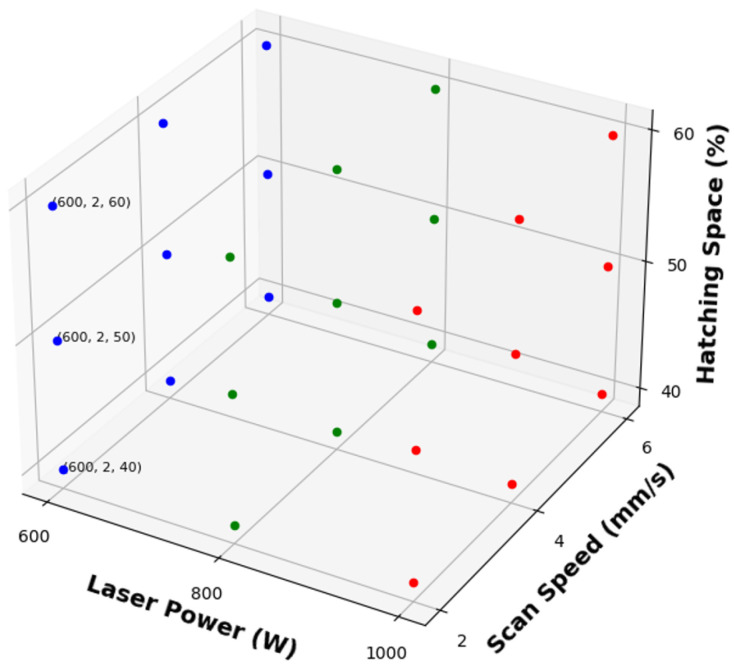
Factorial design of experiments.

In this research, a factorial design of experiments (DOE) is employed, involving three factors, each at three different levels. This methodical approach is designed to thoroughly investigate the interactions and effects of the variables on the outcomes. The chosen factors, critical to the Directed Energy Deposition (DED) process, include laser power (W), scanning speed (mm/s), and hatching space (%). Specifically, the laser power varies between 600 and 1000 watts, the scanning speed ranges from 2 to 6 mm per second, and the hatching space is adjusted from 40% to 60%. These parameters are selected based on their significant influence on the melt pool thermal distribution. A total of 27 experimental runs are conducted to explore the full factorial space, providing a comprehensive understanding of the process dynamics. The schematic detailing these experiments and their configurations is depicted in Figure 2.

**Figure 3:**
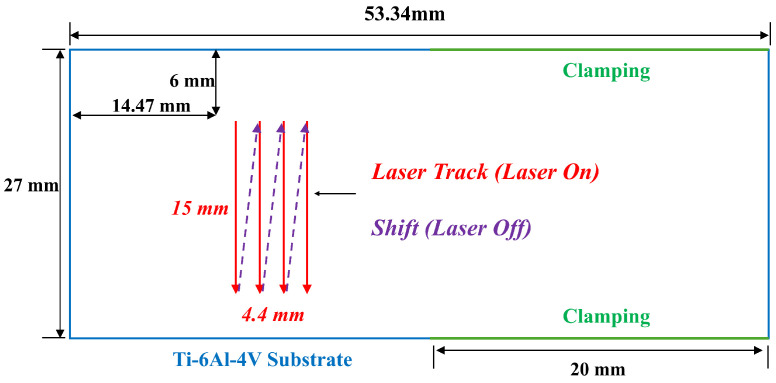
Tool path and simulation setup.

In this research, Ti-6Al-4V is utilized as both the substrate material and the powder. Figure 3 depicts the simulation setup and the laser tool path, featuring a substrate thickness of 6.35 mm. This design incorporates four vertical single laser tracks that run from top to bottom. The total width of the deposit varies from 4.4 mm to 5.6 mm depending on the hatching space, with a length of 15 mm and a thickness of 0.5 mm. The red-colored line indicates that the laser is active, while the purple dashed line signifies that the gantry is moving to the next track and the laser is turned off. In this setup, cantilever clamping, shown in green, extends from the left end to 20 mm. Table 1 details the process parameters for the factorial design of experiments, while Table 2 presents the complete design used for the subsequent multi-physics simulation analysis.

### 2.2. Multi-Physics Simulation

**Figure 4:**
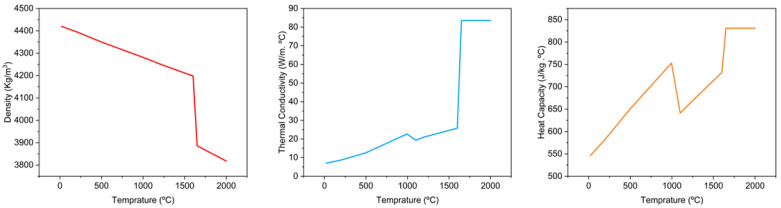
Thermal properties of Ti6Al4V [40].

After designing the experiments, each of the 27 runs was simulated in Abaqus CAE using the AM Modeler plug-in. For the thermal simulation, temperature-dependent material properties of Ti6Al4V were used, as shown in Figure 4.

To perform the calculation of thermal distribution during laser and material deposition, 3D heat conduction equation was employed over the domain shown as T(x,y,z,t) while incorporating appropriate initial and boundary conditions as shown in Equation (1) [41,42].

(1) $$\begin{array}{c} \rho C\frac{\partial T}{\partial t}=\frac{\partial }{\partial x}\left(k\frac{\partial T}{\partial x}\right)+\frac{\partial }{\partial y}\left(k\frac{\partial T}{\partial y}\right)+\frac{\partial }{\partial z}\left(k\frac{\partial T}{\partial z}\right)+Q \end{array}$$

where ρ is density, *C* is specific heat, *T* is temperature, *t* is time, *k* is thermal conductivity, and *Q* is heat flux in the form of laser heat source. To calculate heat loss due to convection, Newton’s law cooling was employed as shown in Equation (2) [41,42].  

(2) $$\begin{array}{c} q_{conv}=h\left(T-T_{env}\right) \end{array}$$

where *h* is convective coefficient which is 30.0 (W/m^2^·K^4^), *T* is shown as temperature at any given time on the surface of the substrate, and $T_{env}$ is room temperature which is 25.0 °C. Heat loss due to radiation is calculated using the Stephen–Boltzmann radiation law as shown in Equation (3) [41,42].

(3) $$q_{rad}=\epsilon \sigma \left(T^{4}-T_{env}^{4}\right)$$

where ϵ is known as emissivity and its value is taken as 0.8, σ represents the Stephen–Boltzmann constant with a value of $5.67\times 10^{-8}$ W/m^2^·K^4^. For body heat flux, Goldaks’s double ellipsoid heat distribution is used as shown in Equation (4) [42,43].

(4) $$Q=\frac{6\sqrt{3}P\eta }{abc\sqrt{\pi }}exp\left(-\frac{3x^{2}}{a^{2}}-\frac{3y^{2}}{b^{2}}-\frac{3{\left(z+V_{s}t\right)}^{2}}{c^{2}}\right)$$

where *P* is power in Watts, η is the efficiency of laser absorption and taken as 0.6, *a* and *b* values are taken as 1 and 2 mm, *c* for both front and back are taken 1 and 2 mm, respectively. $V_{s}$ is the scan speed with which the laser moves in the z-direction.

### 2.3. Data Generation and Extraction

**Figure 5:**
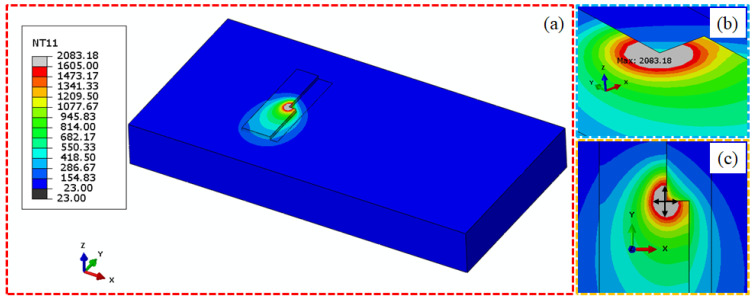
Thermal simulation during material deposition of Run27 as shown in (**a**), maximum temperature value extraction (**b**) and melt pool dimension (**c**).

After solving all the given designs of experiments, data were extracted from each of the ODB files using a Python script. Two types of data were extracted: maximum temperature and melt pool dimensions. Therefore, separate scripts were used for each type. For example, run number 27 is shown in Figure 5a during material deposition and analysis.

For the maximum temperature, the highest temperature value was extracted for each increment from each frame as shown in Figure 5b during Run27 simulation. The same concept was applied to extract and calculate the melt pool dimensions. For each successful increment solved during the analysis, all nodal locations in all directions with values equal to or above 1605 °C were extracted. Once extracted for the specific increment, the location with the highest value in length was subtracted from the location with the lowest value of length, essentially providing the relevant dimension. The same method was employed for extracting the melt pool length, width, and depth as shown in Figure 5c.

### 2.4. Machine Learning Models

After extracting data from finite element simulations, four machine learning algorithms—Extreme Gradient Boosting (XGBoost), Long Short-Term Memory (LSTM), Bidirectional Long Short-Term Memory (Bi-LSTM), and Gated Recurrent Units (GRUs)—are prepared to build a surrogate model for predicting the thermal history and dimensions of the melt pool. Both the accuracy and computational time of these algorithms are considered to construct a robust machine learning-based surrogate model. The subsequent sections describe each algorithm’s advantages and mathematical concepts.

#### 2.4.1. Extreme Gradient Boosting (XGBoost)

XGBoost is recognized as one of the most effective applications of gradient-boosted decision trees [44]. Explicitly proposed to augment memory utilization and leverage hardware computational power, XGBoost significantly reduces accomplishment time while enhancing performance compared to other ML algorithms. The core concept of boosting involves sequentially constructing sub-trees from an original one, where each successive tree aims to lessen the errors of the preceding one. This iterative method updates the prior residuals, thereby minimizing the error of the cost function. Let us assume a dataset illustrated as [44]

(5) $$D=\{\left(x_{i},y_{i}\right)|x_{i}\in R^{m},y_{i}\in R\}.$$

Here, *m*, $x_{i}$, and $y_{i}$ are the feature dimensions and the samples’ (*i*) responses, respectively. In addition, *n* represents the sample number (|D|=n). The forecasted output ($y_{i}$) for an entry (*i*) is as follows [44]:

(6) $$y^{i}=\sum_{k=1}^{K}f_{k}\left(x_{i}\right),\;f_{k}\in F.$$

In the above Equation, $f_{k}$ represents a standalone tree within *F*, and $f_{k}\left(x_{i}\right)$ indicates the projected result from the *i*th trial and *k*th tree. The objective function (L) is written as [44]

(7) $$L=\sum_{i=1}^{n}l\left(y_{i},{\hat{y}}^{i}\right)+\sum_{k=1}^{K}\Omega \left(f_{k}\right).$$

By minimizing the actual function (L), the regression tree model functions ($f_{k}$) are attained. The loss function ($l(y_{i},{\hat{y}}_{i})$) assesses the differentiation between estimated (${\hat{y}}_{i}$) and real outputs ($y_{i}$). So, the term Ω is applied to prevent the overfitting issue by correcting the model intricacy, explained as [44]

(8) $$\Omega \left(f_{k}\right)=\gamma T+\frac{1}{2}\lambda {∥w∥}^{2}.$$

Here, γ as well as λ are regularization factors, *T* and *w* are designated as the number and score of the leaf, respectively. A Taylor series expansion with the second degree can be applied to estimate the target function. We assume that $I_{j}=\left\{i|q\left(x_{i}\right)=j\right\}$ is an insistence set of leaf *j* having q(x) as a permanent configuration. The optimum weights $w_{j}^{*}$ of *j* and the subsequent quantity are estimated as [44]

(9) $$w_{j}^{*}=-\frac{g_{j}}{h_{j}+\lambda }.$$

(10) $$L^{*}=-\frac{1}{2}\sum_{j=1}^{T}\frac{{\left(\sum_{i\in I_{j}}g_{i}\right)}^{2}}{\sum_{i\in I_{j}}h_{i}+\lambda }+\lambda T.$$

Here, the first- and second-order gradients for L are represented by $g_{i}$ and $h_{i}$, respectively. L can be applied as a quality index of the tree (*q*) so that the model is outstanding if the score is lower. It is not possible to consider the whole tree structure at a time. An excellent algorithm should resolve the challenge by initiating from an individual leaf and iteratively increasing branches. We assume that the right and left instance nodes are represented by $I_{R}$ as well as $I_{L}$, respectively. Considering $I=I_{R}\cup I_{L}$, the loss reduction can be written as following the split [44]:

(11) $$L_{\mathrm{split}}=\frac{1}{2}\left[\frac{{\left(\sum_{i\in I_{L}}g_{i}\right)}^{2}}{\sum_{i\in I_{L}}h_{i}+\lambda }+\frac{{\left(\sum_{i\in I_{R}}g_{i}\right)}^{2}}{\sum_{i\in I_{R}}h_{i}+\lambda }-\frac{{\left(\sum_{i\in I}g_{i}\right)}^{2}}{\sum_{i\in I}h_{i}+\lambda }\right]-\gamma .$$

The XGBoost model employs numerous simple trees and assigns scores to leaf nodes during the splitting process.

#### 2.4.2. Long Short-Term Memory (LSTM)

LSTM networks, an advanced type of recurrent neural networks, effectively address long-range dependencies in sequence data, crucial in scenarios like directed energy deposition processes. Characterized by three distinct gates—input, forget, and output—LSTMs manage information flow, selectively retaining or discarding data to precisely learn dependencies. The input state ($i_{t}$) decides which new information to incorporate into the cell state ($c_{t}$) and candidate state (${\tilde{c}}_{t}$), enabling the model to update its memory with relevant data. The forget gate ($f_{t}$) selectively removes irrelevant information from the cell state to maintain the model’s focus on pertinent data through time. The output gate ($o_{t}$) controls the flow of information from the cell state to the next layer or time step, determining what part of the hidden state ($h_{t}$) is used to compute the output and pass to next iteration.

**Figure 6:**
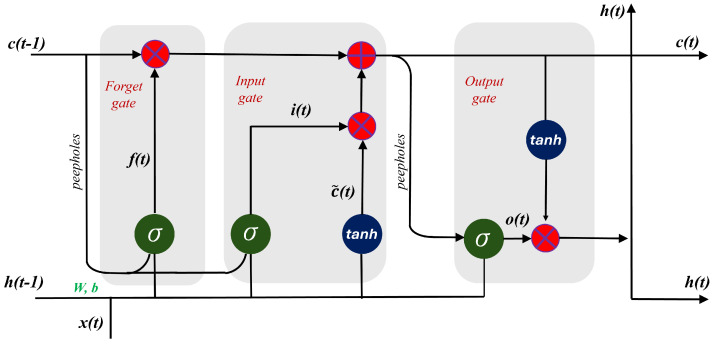
Architecture of LSTM algorithm.

This architecture mitigates gradient vanishing and exploding issues, enhancing robustness and accuracy in predictive models and making LSTM ideal for capturing complex thermal and mechanical interactions in additive manufacturing. The LSTM architecture is shown in Figure 6. The operator ‘×’ denotes pointwise multiplication, and ’+’ denotes pointwise addition. The mathematical framework of LSTMs is presented in [45].

Forget gate:

(12) $$f_{t}=\sigma \left(W_{fh}h_{t-1}+W_{fx}x_{t}+P_{f}\cdot c_{t-1}+b_{f}\right)$$

Input gate:

(13) $$i_{t}=\sigma \left(W_{ih}h_{t-1}+W_{ix}x_{t}+P_{i}\cdot c_{t-1}+b_{i}\right)$$

(14) $${\tilde{c}}_{t}=\tanh\left(W_{ch}h_{t-1}+W_{cx}x_{t}+b_{\tilde{c}}\right)$$

(15) $$c_{t}=f_{t}\cdot c_{t-1}+i_{t}\cdot {\tilde{c}}_{t}$$

Output gate:

(16) $$o_{t}=\sigma \left(W_{oh}h_{t-1}+W_{ox}x_{t}+P_{o}\cdot c_{t}+b_{o}\right)$$

(17) $$h_{t}=o_{t}\cdot \tanh\left(c_{t}\right)$$

**Figure 7:**
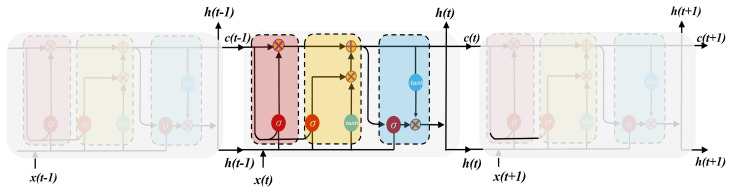
Series of LSTM architecture.

Here, $W_{f}$, $W_{i}$, $W_{c}$, and $W_{o}$ are the weights of each input. The $x_{t}$, $h_{t}$, and $y_{t}$ are represented as input, hidden state (recurrent information), and output concerning time. Furthermore, the $f_{t}$ is the forget cell starting from 0, $P_{f}$, $P_{i}$, and $P_{o}$ are the peephole weights for $f_{t}$, input, and output gates. The $c_{t}$ denotes the LSTM cell state, and $b_{i}$, $b_{f}$, $b_{\tilde{c}}$, and $b_{o}$ are the biases. Figure 7 shows the architecture of the series of LSTM structures.

#### 2.4.3. Bidirectional Long Short-Term Memory (Bi-LSTM)

**Figure 8:**
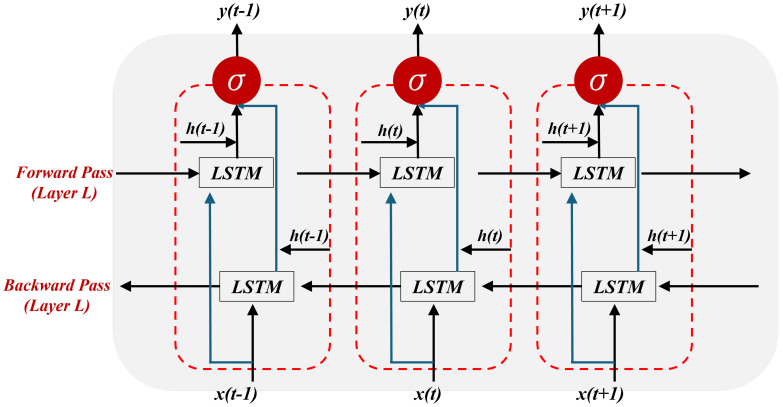
Architecture of Bi-LSTM algorithm.

Bi-LSTM networks enhance traditional LSTM by processing data both forwards and backwards, enriching sequence context understanding. This dual-path approach not only boosts predictive accuracy in tasks like outcome prediction in directed energy deposition but also captures nuanced temporal dynamics from both past and future contexts. Despite their increased computational demands and potential for overfitting with small datasets, Bi-LSTMs remain valuable for thoroughly analyzing thermal and mechanical properties in AM. Leveraging LSTM strengths, they effectively manage long-term dependencies and mitigate gradient issues, providing a robust model for complex material behaviors. The Bi-LSTM architecture is defined in Figure 8, and the LSTM block within this architecture follows the structure shown in Figure 6. The mathematical expression is given in [45].

(18) $$f_{t}^{L}=\sigma \left(W_{fh}^{L}h_{t-1}^{L}+W_{fx}^{L}h_{t}^{L-1}+b_{f}^{L}\right)$$

(19) $$i_{t}^{L}=\sigma \left(W_{ih}^{L}h_{t-1}^{L}+W_{ix}^{L}h_{t}^{L-1}+b_{i}^{L}\right),$$

(20) $${\tilde{c}}_{t}^{L}=\tanh\left(W_{\tilde{c}h}^{L}h_{t-1}^{L}+W_{\tilde{c}x}^{L}h_{t-1}^{L}+b_{\tilde{c}}^{L}\right),$$

(21) $$c_{t}^{L}=f_{t}^{L}\cdot c_{t-1}^{L}+i_{t}^{L}\cdot {\tilde{c}}_{t}^{L},$$

(22) $$o_{t}^{L}=\sigma \left(W_{oh}^{L}h_{t-1}^{L}+W_{ox}^{L}h_{t-1}^{L}+b_{o}^{L}\right)$$

(23) $$h_{t}^{L}=o_{t}^{L}\cdot \tanh\left(c_{t}^{L}\right).$$

(24) $$y_{t}=W_{hy}^{\to }h_{t}+W_{hy}^{\leftarrow }h_{t}+b_{y}$$

Here, $h_{t}^{L}$ represents the output of the hidden state in the (*L*)th layer at time *t*. Equation (24) shows the output of architecture, where $W_{hy}^{\to }$ denotes the weight of the forward pass, $W_{hy}^{\leftarrow }$ indicates the weight of the backward pass, and $b_{y}$ signifies the bias of the output.

#### 2.4.4. Gated Recurrent Units (GRUs)

**Figure 9:**
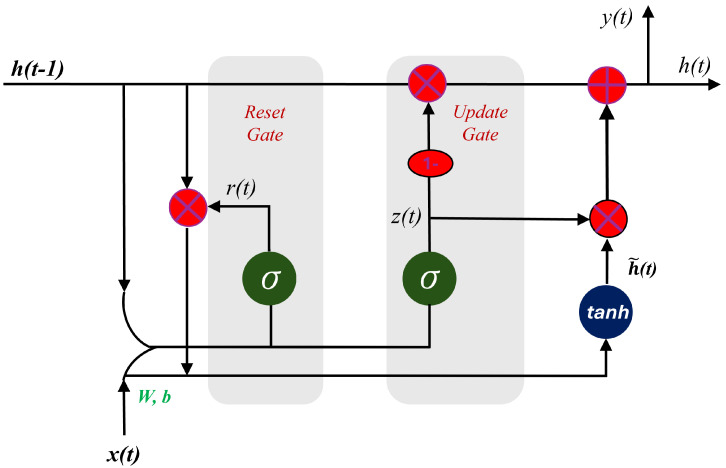
Architecture of GRU algorithm.

GRUs offer a streamlined alternative to LSTMs and Bi-LSTMs, ideal for modeling thermal histories in directed energy deposition. By employing just two gates—the reset and update gates—GRUs enhance computational efficiency and reduce model complexity, making them well-suited for scenarios with limited data or computational resources. The reset gate determines how much past information to forget. In contrast, the update gate decides how much of the current input should be incorporated, allowing the model to handle time dependencies dynamically. Although GRUs may struggle with extremely long dependencies, their ability to efficiently process sequential data without significant computational overhead keeps them highly relevant for improving predictive models in AM. Based on Figure 9, the following mathematical model has been proposed for GRU [45]:

Reset gate:

(25) $$r_{t}=\sigma \left(W_{rh}h_{t-1}+W_{rx}x_{t}+b_{r}\right),$$

Update gate:

(26) $$z_{t}=\sigma \left(W_{zh}h_{t-1}+W_{zx}x_{t}+b_{z}\right),$$

(27) $${\tilde{h}}_{t}=\tanh\left(W_{\tilde{h}h}\left(r_{t}\cdot h_{t-1}\right)+W_{\tilde{h}x}x_{t}+b_{\tilde{h}}\right),$$

(28) $$h_{t}=\left(1-z_{t}\right)\cdot h_{t-1}+z_{t}\cdot {\tilde{h}}_{t}.$$

Here, $W_{r}$, $W_{z}$, and $W_{\tilde{h}h}$ are the weights, and $b_{r}$ and $b_{z}$ are the biases.

#### 2.4.5. Model Evaluation

In the model evaluation part, the performance of the surrogate model is assessed using several statistical metrics to ensure accuracy and reliability. These metrics include R-squared (R²), which measures the proportion of variance in the dependent variable that is predictable from the independent variables; Root Mean Square Error (RMSE), which provides the standard deviation of the prediction errors or residuals; and Mean Absolute Error (MAE), which represents the average magnitude of the errors in a set of predictions, without considering their direction. The mathematical formulas are presented as follows:

(29) $$R^{2}=1-\frac{\sum_{i=1}^{n}{\left(y_{i}-{\hat{y}}_{i}\right)}^{2}}{\sum_{i=1}^{n}{\left(y_{i}-\bar{y}\right)}^{2}}$$

(30) $$RMSE=\sqrt{\frac{1}{n}\sum_{i=1}^{n}{\left(y_{i}-{\hat{y}}_{i}\right)}^{2}}$$

(31) $$MAE=\frac{1}{n}\sum_{i=1}^{n}\left|y_{i}-{\hat{y}}_{i}\right|$$

Here, $y_{i}$ are the observed values, ${\hat{y}}_{i}$ are the predicted values, and $\bar{y}$ is the mean of the observed values. Additionally, the computation time of the training model is also considered a factor in evaluating model performance.

## 3. Results and Discussion

### 3.1. Data Pre-Processing and Model Training

**Figure 10:**
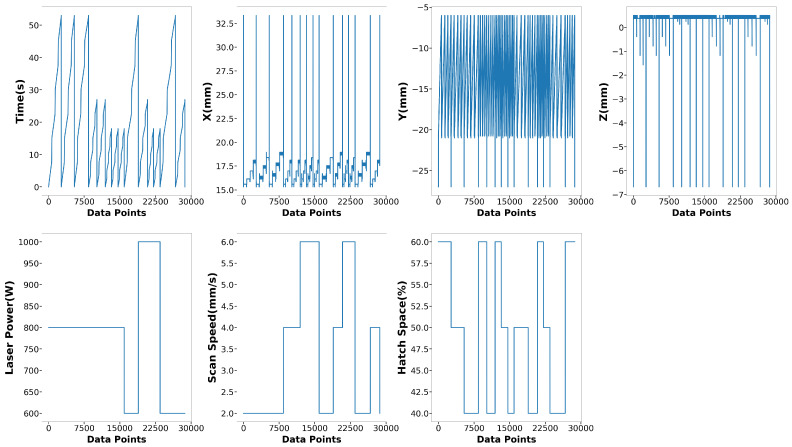
Training Features of melt pool peak temperature model.

**Figure 11:**
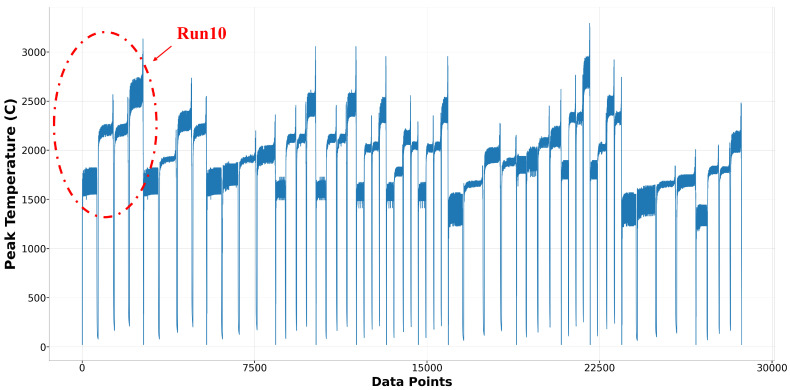
Training Label of melt pool peak temperature model.

**Figure 12:**
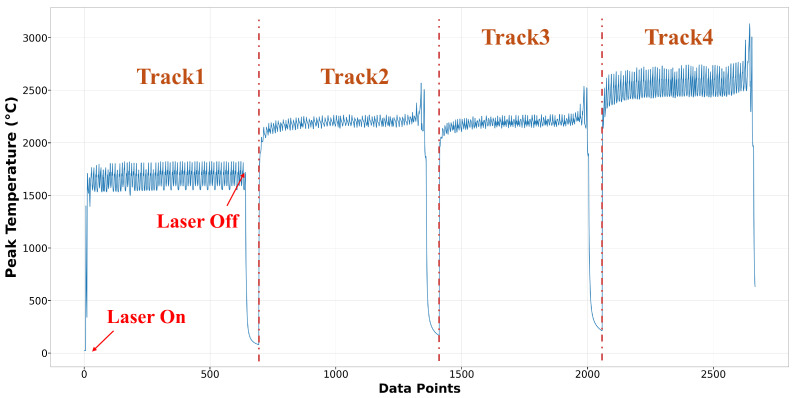
Training label of Run10 in melt pool peak temperature model.

The data used to build the surrogate models for melt pool peak temperature and melt pool dimension in this research originated from 27 runs of multi-physics modeling, employing a three-level, three-factor factorial design of experiments. A total of 54,956 data points were extracted. For the melt pool peak temperature model, data points that did not reach the melting point of Ti-6Al-4V (1605 °C) or exceeded (3200 °C) were excluded. The vaporization point of Ti-6Al-4V is 3040 °C, but melt pool peak temperatures occasionally exceed this threshold. To accommodate most conditions during deposition, temperatures above the vaporization point were also considered. After cleaning, the dataset contained 38,867 peak temperature points, with 28,683 allocated for training and 10,184 for testing. The training and testing sets accounted for 73.8% and 26.2%, respectively. Figure 10 displays the training features: time, x position, y position, z position, laser power, scanning speed, and hatch space. Figure 11 depicts the training label for melt pool peak temperature. A detailed and clear description of the training label is shown in Figure 12. The peak temperature dramatically increases when the laser is on and drops when it is off. Each run consists of four tracks, and fluctuations occur during the movement in each track.

**Figure 13:**
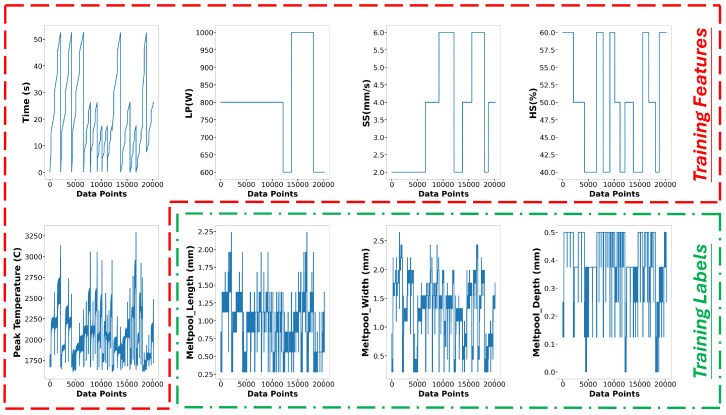
Training features and labels of melt pool dimension model.

Regarding the melt pool dimension model, 27,772 data points were collected because data on melt pool dimensions are extracted only when the border of the melt pool exceeds 1605 °C, as described in Section 2. These points are divided into 20,182 (72.6%) for training and 7590 (27.4%) for testing. The features and labels are shown in Figure 13. In the melt pool dimension model, time (s), laser power (W), scanning speed (mm/s), hatching space (%), and peak temperature (°C) are considered as features, while melt pool length, width, and depth (mm) are considered as labels. After removing outliers, such as extremely high and low thermal histories, 19 runs of data remain: 14 runs are designated for training and 5 runs for testing. To mitigate the impact of disproportionately large values among the process parameters and training features, normalization is applied in the data pre-processing stage. The details of the data for the two surrogate models, the melt pool peak temperature model, and the melt pool dimension model are described in Table 3.

With regard to model training, the grid search method is applied to find the proper hyperparameters. For the XGBoost algorithm, the tree depth is set to five to avoid overfitting, with a learning rate of 0.01 to ensure steady convergence. The objective is defined as ‘reg:squarederror’ to minimize squared errors in regression tasks. L1 regularization (reg_alpha) is applied at 0.01 to promote parameter sparsity, and L2 regularization (reg_lambda) is set at 1 to reduce weight extremes. Both subsample and colsample_bytree are maintained at 0.8, allowing the model to learn from 80% of data and features, respectively, to prevent overfitting. The evaluation metric used is ‘rmse’, measuring prediction accuracy. Training involves 10,000 rounds, optimizing learning against computational demands. In terms of RNN algorithms, the hyperparameters for all LSTM, Bi-LSTM, and GRU algorithms are unified to ensure a fair comparison among the models. The sequence length of data is set to 10, batch size to 64, dropout rate to 0.25, hidden dimension to 100, number of layers to 2, learning rate to 0.001, and number of epochs to 100.

### 3.2. Melt Pool Peak Temperature Model

In this section, four different algorithms—XGBoost, Bi-LSTM, LSTM, and GRU—are applied to this research. To compare the pros and cons of tree-based versus RNN algorithms, the predicted results by XGBoost and Bi-LSTM are presented together in one figure. In terms of comparing the complexity of RNN algorithms, the results of LSTM and GRU are displayed together in another figure. Additionally, two specific runs, Run1 (Laser Power: 600W, Scanning Speed: 2 mm/s, Hatching Space: 60%) and Run27 (Laser Power: 1000 W, Scanning Speed: 6 mm/s, Hatching Space: 40%), are extracted and analyzed to facilitate a detailed comparison and enhance clarity. A comprehensive comparison of predictions by four algorithms is also included in this section.

**Figure 14:**
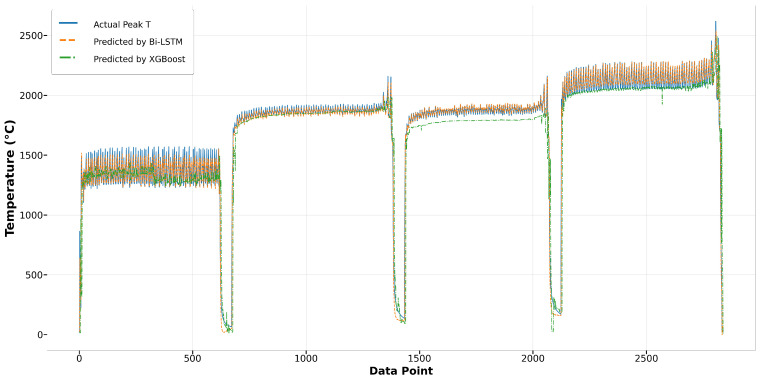
Run1: actual peak temperature versus prediction by Bi-LSTM and XGBoost.

**Figure 15:**
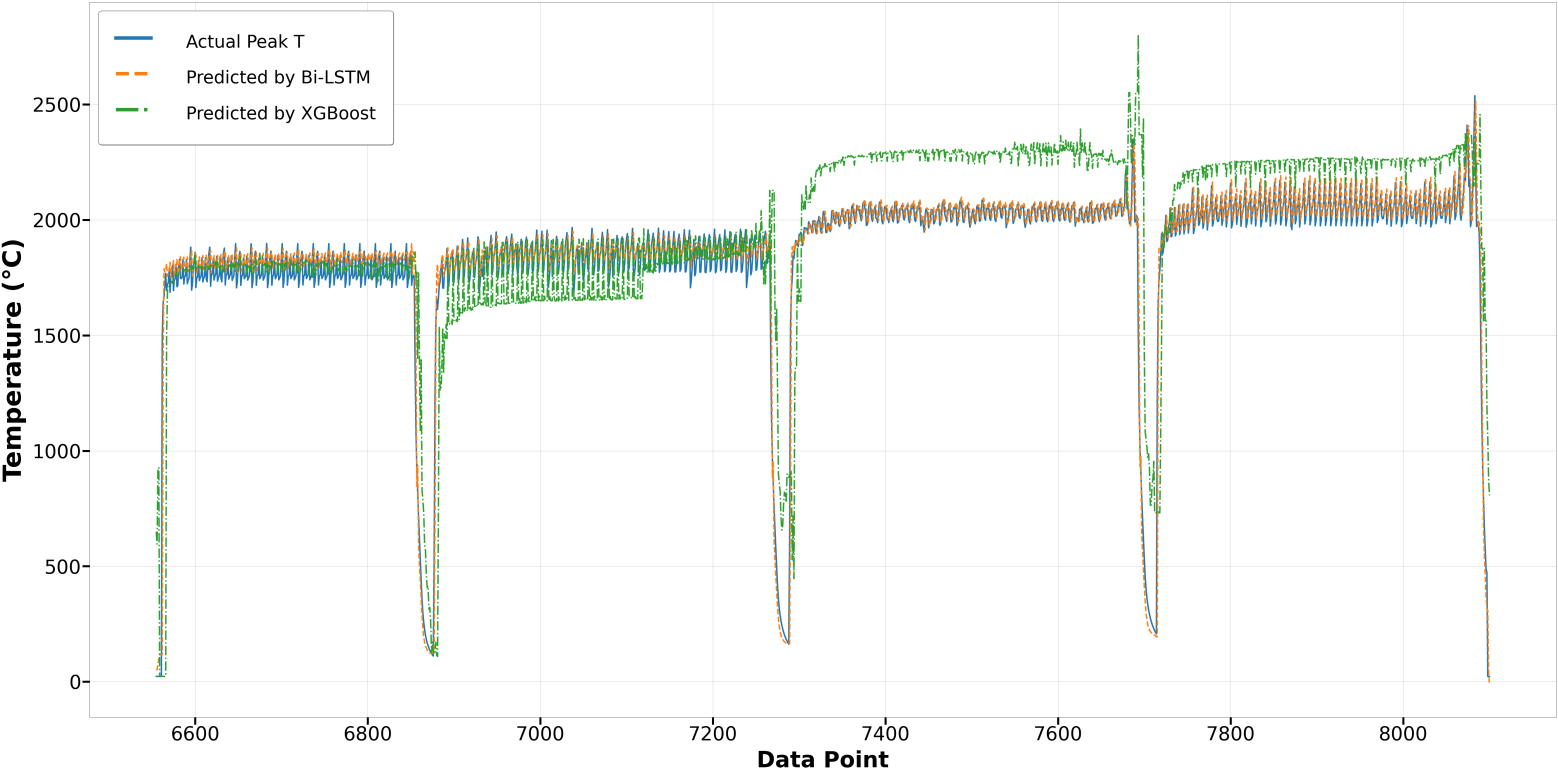
Run27: actual peak temperature versus prediction by Bi-LSTM and XGBoost.

**Figure 16:**
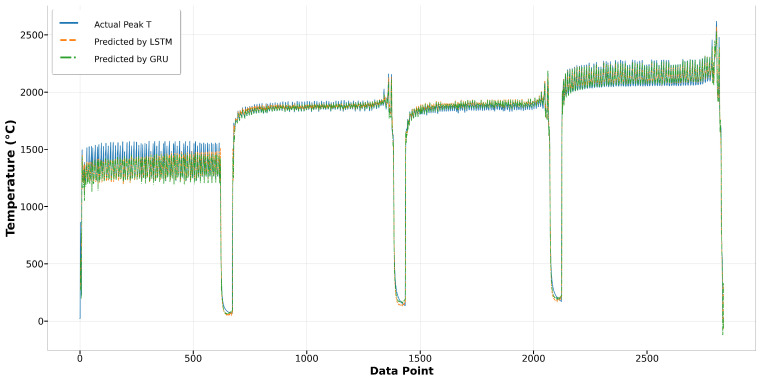
Run1: actual peak temperature versus prediction by LSTM and GRU.

**Figure 17:**
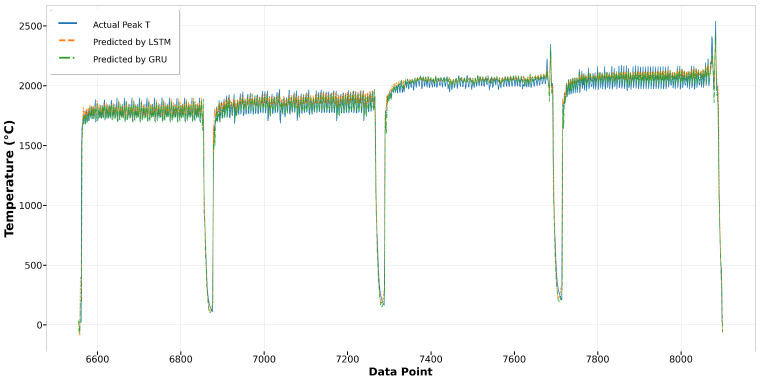
Run27: Actual peak temperature versus prediction by LSTM and GRU.

Figure 14 depicts the comparison of Run1 among actual values and predicted values by Bi-LSTM and XGBoost. It shows that the melt pool peak temperatures predicted by Bi-LSTM closely match the actual peak temperatures. The results from XGBoost also demonstrate reasonably good prediction performance. However, in Run27, the predictions by XGBoost significantly deviate from the actual peak temperatures, especially in the second and third tracks, where the predictions have more fluctuation and are higher than the actual values. In contrast, the results from Bi-LSTM closely align with the actual values, demonstrating the robustness of the model built using the Bi-LSTM algorithm, as shown in Figure 15. In terms of the other two algorithms, LSTM and GRU, both achieve good predictions that closely fit the actual values. In the first track of Run1, both predictions are slightly lower than the actual values, yet the remaining predictions demonstrate good performance, as depicted in Figure 16. In Run27, shown in Figure 17, except for the fourth track, where the predictions are slightly lower than the actual values, most of the results closely match the actual values.

**Figure 18:**
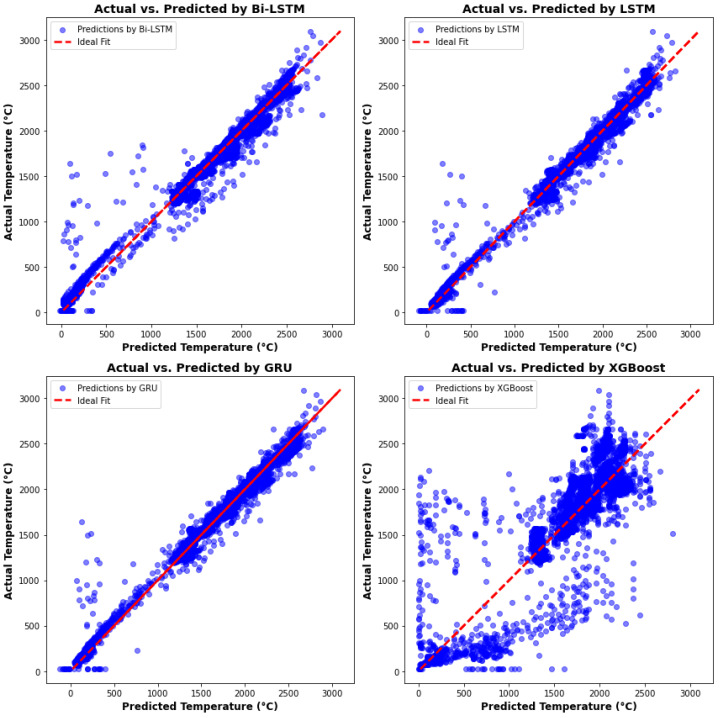
Actual peak temperature with predictions from four algorithms.

All predicted temperatures versus actual temperatures scatter plots are shown in Figure 18. It demonstrates that the predicted values by XGBoost are relatively less accurate than those produced by RNN algorithms. Most of the predicted results by RNN algorithms closely match the red line, which has a slope of one, indicating that the predictions are both accurate and robust. To compare the performance of the four algorithms, Table 4 reveals that the Bi-LSTM model has the highest accuracy, longest computational time, and greatest memory usage. Although XGBoost performs well in terms of computational time and memory usage, its accuracy is not robust enough to predict melt pool peak temperatures reliably. The accuracy of the LSTM and GRU models is similar; however, the computational time and memory usage of the GRU model are lower than those of the LSTM model by 20.7% and 5.4%, respectively. In conclusion, the Bi-LSTM model provides the most accurate results, while the GRU model offers comparable accuracy with lower computational time and memory usage.

### 3.3. Melt Pool Geometry Model

In this section, three surrogate models are presented: melt pool length, width, and depth, respectively. To clarify the comparison, results from Run23 and Run14 are extracted for discussion. Additionally, the overall results of the four algorithms are compared using scatter plots and a comprehensive table in the subsequent contents.

#### 3.3.1. Melt Pool Length Model

**Figure 19:**
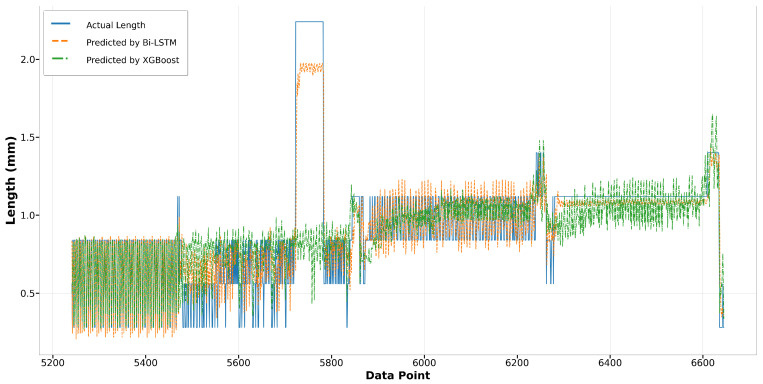
Run14: Actual length versus prediction by Bi-LSTM and XGBoost.

**Figure 20:**
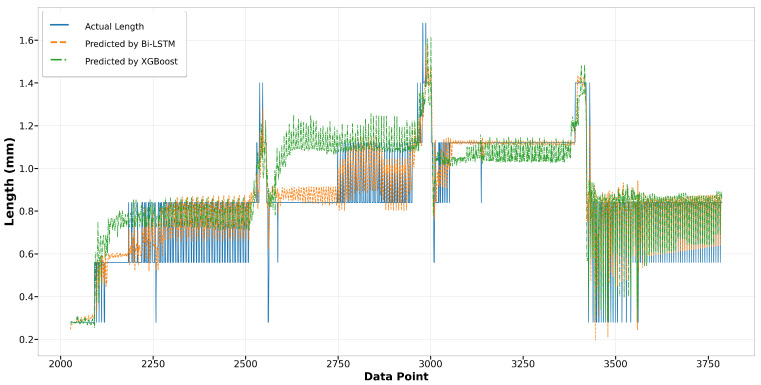
Run23: Actual length versus prediction by Bi-LSTM and XGBoost.

In Run14 and Run23, the Bi-LSTM model consistently outperforms the XGBoost model in predicting melt pool length. As depicted in Figure 19, the Bi-LSTM model demonstrates superior accuracy in predicting higher melt pool lengths, particularly for data points from 5700 to 5800. Moreover, towards the end of Run14, from data points 6300 to 6700, the Bi-LSTM model shows significantly less fluctuation compared to the XGBoost model, indicating its enhanced stability under varying conditions. In Figure 20, although the XGBoost model accurately predicts the melt pool lengths for data points from 3400 to 3750, the overall performance of the Bi-LSTM model remains more consistent and aligned with the actual length.

**Figure 21:**
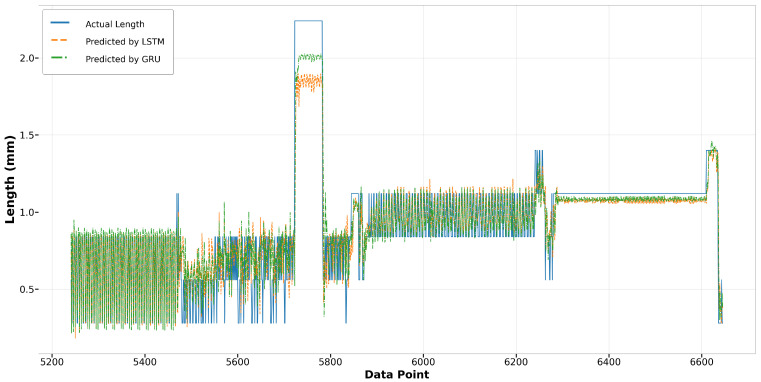
Run14: actual length versus prediction by LSTM and GRU.

**Figure 22:**
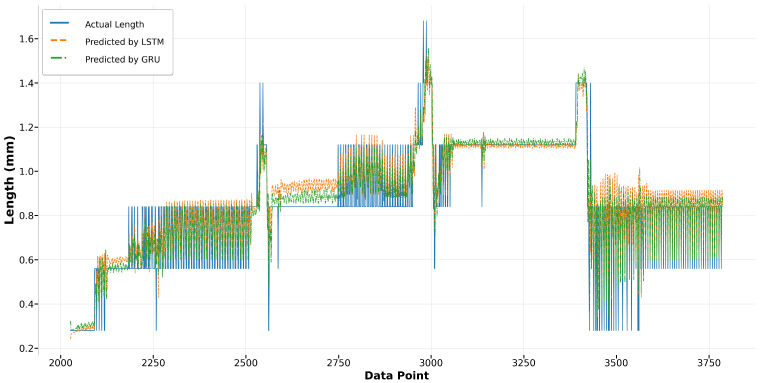
Run23: actual length versus prediction by LSTM and GRU.

Regarding the LSTM and GRU models, the GRU model exhibits less error in predicting longer melt pool lengths, as evident in Figure 21 for data points from 5700 to 5800. Throughout the remainder of Run14, both models achieve commendable accuracy in fitting the actual length. In Run23, despite both models displaying a similar trend in capturing the actual values, the LSTM model exhibits greater deviations from the actual lengths compared to the GRU model, as illustrated in Figure 22. This result suggests that while the LSTM model is generally reliable, the GRU model may offer better consistency and precision under certain conditions.

**Figure 23:**
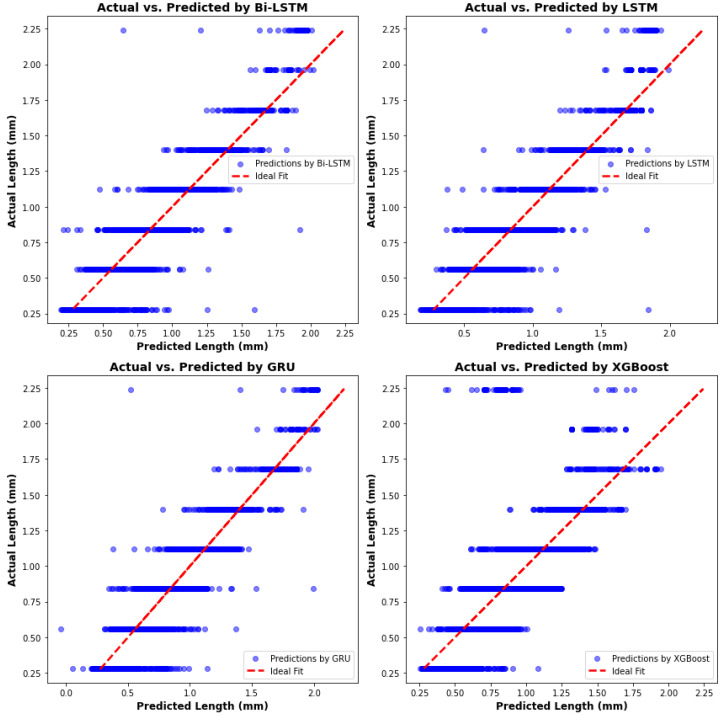
Actual length versus predictions from four algorithms.

The comparison of overall predictions among four algorithms is presented in a scatter plot. Figure 23 demonstrates that the melt pool lengths predicted by the RNN algorithms are more accurate than those predicted by the XGBoost algorithm. Notably, when the melt pool length exceeds 2 mm, predictions from the XGBoost model deviate significantly from the ideal fit, resulting in decreased accuracy. Table 5 summarizes the evaluation and comparative analysis of the melt pool length models. Although the XGBoost algorithm impresses with its computation time and memory usage, its accuracy needs improvement. In terms of RNN algorithms, the GRU and Bi-LSTM models perform better. In particular, the GRU model not only achieves the highest R-square value but also requires the least computation time and memory usage among all RNN algorithms. Compared to the Bi-LSTM model, the GRU model’s computation time and memory usage are lower by 44% and 51%, respectively, making it the most suitable candidate for predicting melt pool length in this research.

#### 3.3.2. Melt Pool Width Model

**Figure 24:**
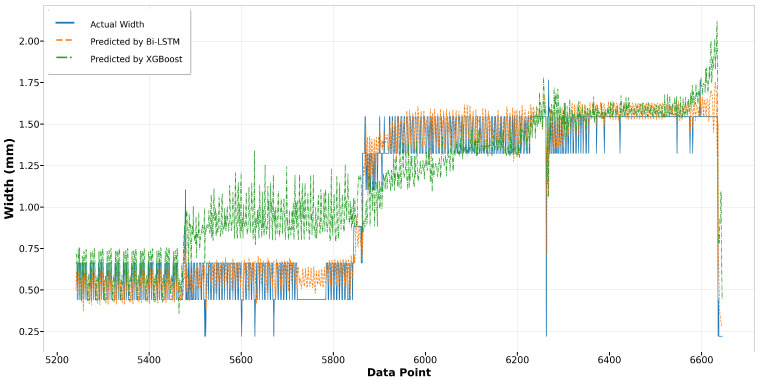
Run14: actual width versus prediction by Bi-LSTM and XGBoost.

**Figure 25:**
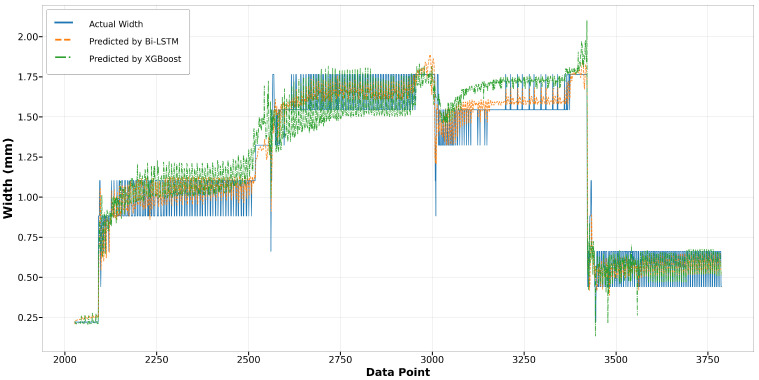
Run23: actual width versus prediction by Bi-LSTM and XGBoost.

In the melt pool width model, Figure 24 and Figure 25 illustrate the predictions made by the Bi-LSTM and XGBoost algorithms for Run14 and Run23, respectively. Those scatter plots show a notable variance in accuracy between the algorithms. For Run14, particularly from data point 5500 to 5900, and in Run23 from data point 3100 to 3400, the predictions by the XGBoost model significantly exceed the actual width, highlighting its lower accuracy compared to the Bi-LSTM model. The Bi-LSTM model more consistently aligns with the actual measurements, particularly in complex segments where the melt pool width fluctuates.

**Figure 26:**
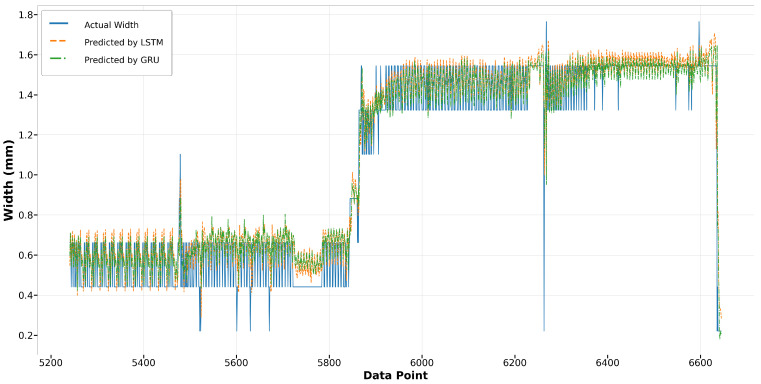
Run14: actual width versus prediction by LSTM and GRU.

**Figure 27:**
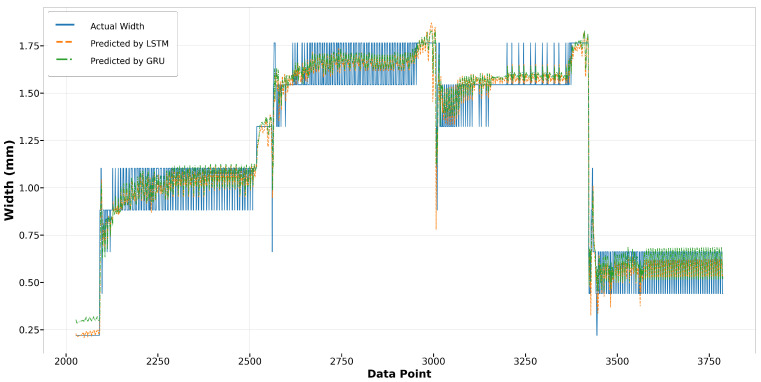
Run23: actual width versus prediction by LSTM and GRU.

Figure 26 and Figure 27 showcase the performance of the LSTM and GRU models for Run14 and Run23, respectively. Both models exhibit similar trends and achieve commendable accuracy in fitting the actual widths in Run14, with the GRU model slightly outperforming the LSTM. Notably, in Run23, while neither model perfectly replicates the fluctuation observed in the actual width measurements, they successfully capture the broader trends. The GRU model consistently demonstrates a slight edge over the LSTM in terms of alignment with the actual data across both runs, indicating its robustness in modeling the melt pool width.

**Figure 28:**
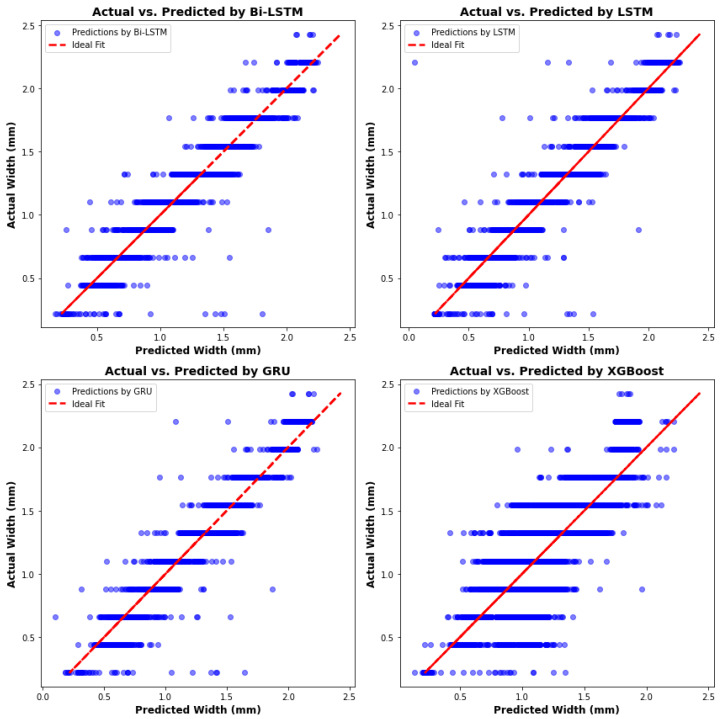
Actual width versus predictions from four algorithms.

The overall predictive performance of four algorithms is displayed in a scatter plot for comparison, as shown in Figure 28. The RNN algorithms, particularly Bi-LSTM and GRU, exhibit superior performance in predicting sequential data such as melt pool width, evidenced by their close alignment with the ideal fit line. Both models display similar commendable accuracy, effectively capturing the sequential dependencies within the data. In contrast, predictions by the XGBoost algorithm are notably more dispersed, indicating less accuracy. This dispersion becomes especially pronounced when the actual width exceeds 2 mm, where XGBoost predictions significantly deviate from the ideal fit. Table 6 summarizes the comparison among all algorithms, highlighting that the Bi-LSTM model achieves the highest R-square value. However, the GRU model offers comparable accuracy with lower computation time and memory usage—40% and 51% less than the Bi-LSTM model, respectively—demonstrating its greater robustness.

#### 3.3.3. Melt Pool Depth Model

**Figure 29:**
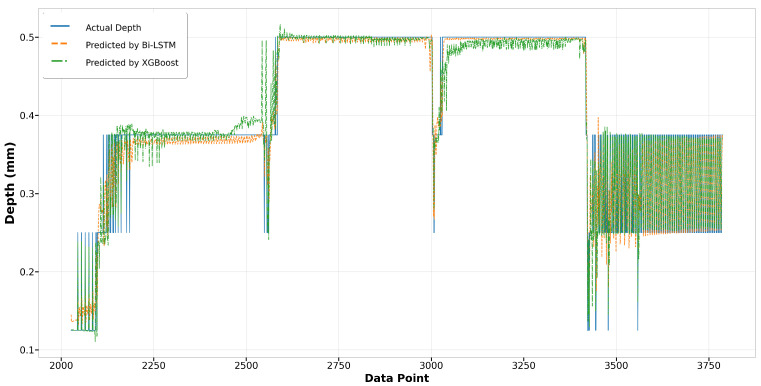
Run14: actual depth versus prediction by Bi-LSTM and XGBoost.

**Figure 30:**
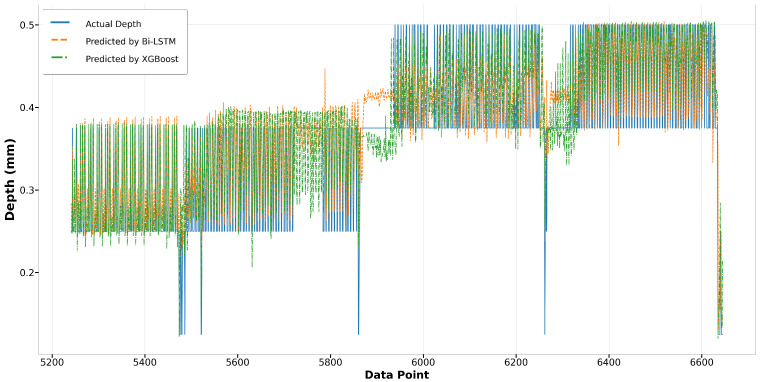
Run23: actual depth versus prediction by Bi-LSTM and XGBoost.

In the melt pool depth model, the XGBoost model displays a surprising parity with the Bi-LSTM model in terms of performance in Run14, especially noticeable at the start where XGBoost surpasses Bi-LSTM in accuracy, as shown in Figure 29. In contrast, during Run23 as depicted in Figure 30, although the overall trends of both models align closely with the actual depth measurements, the XGBoost predictions show greater deviations from the actual values, suggesting less consistency compared to the Bi-LSTM model. This indicates that while XGBoost can match the performance of Bi-LSTM in certain scenarios, its performance can be less reliable in others.

**Figure 31:**
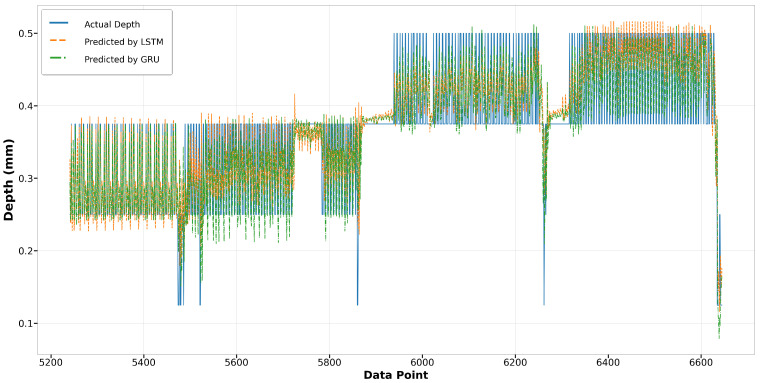
Run14: actual depth versus prediction by LSTM and GRU.

**Figure 32:**
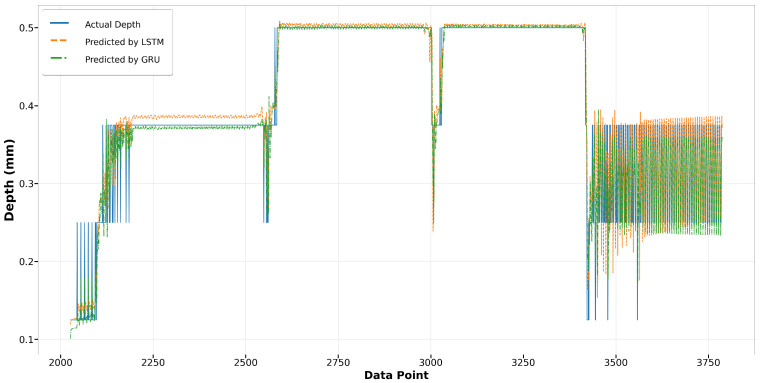
Run23: actual depth versus prediction by LSTM and GRU.

Regarding the LSTM and GRU models, their performance in predicting melt pool depth is commendably consistent, exhibiting similar trends. Both models closely align with the actual values, demonstrating their effectiveness in capturing sequential data characteristics. In Run14, although the predictions start slightly below the actual values, both LSTM and GRU adjust quickly and maintain a good match throughout the data range, as shown in Figure 31. Run23 shows a slight divergence in the predictions from both models, especially in the latter half, where the LSTM model exhibits more deviation than the GRU model, yet both still maintain a general adherence to the trend of actual depth values, as illustrated in Figure 32.

**Figure 33:**
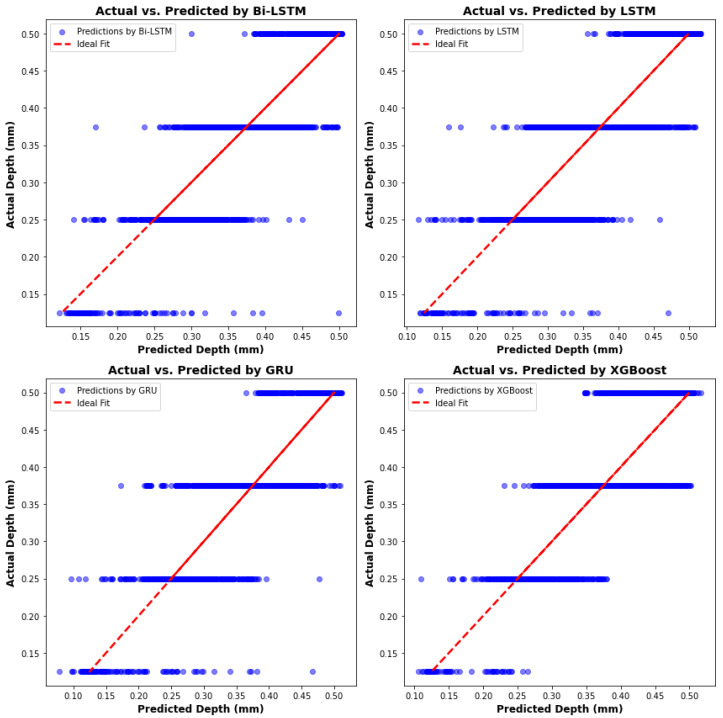
Actual depth versus predictions from four algorithms.

The scatter plots of predictions by all four algorithms are presented in Figure 33. Unlike the melt pool peak temperature and other geometric models, no single model exhibits particularly strong performance. All models deviate from the ideal fit, especially when predicting maximum and minimum melt pool depths. For a more comprehensive comparison and analysis, Table 7 reveals that the XGBoost model has the shortest computation time and lowest memory usage, but relatively lower accuracy. Additionally, the GRU model boasts the highest R-square value and has lower computation time and memory usage—29% and 50% less, respectively, compared to the Bi-LSTM model—highlighting the reliability and robustness of the GRU model.

## 4. Conclusions

This study developed a recurrent neural network (RNN)-based surrogate model to predict melt pool characteristics, such as peak temperature, length, width, and depth, in directed energy deposition (DED) processes. By integrating a three-level, three-factor design of experiments and multi-physics simulation data into an LSTM, Bi-LSTM, and GRU framework, the model demonstrates exceptional predictive accuracy for sequential melt pool data under varied processing conditions. The research also presents a comprehensive evaluation and comparative analysis of surrogate models built with different algorithms. Key contributions of this research include:

- **Robust Model Architecture:** Employed advanced RNN architectures—LSTM, Bi-LSTM, and GRU—to effectively capture the sequential and dynamic behavior of melt pools in DED processes.
- **High Predictive Accuracy:** Achieved an R-square of 0.983 for melt pool peak temperature predictions using the Bi-LSTM algorithm. Demonstrated superior performance in melt pool geometry predictions:
  - Melt pool length: R-square of 0.903 with the GRU algorithm.
  - Melt pool width: R-square of 0.952 with the Bi-LSTM algorithm.
  - Melt pool depth: R-square of 0.885 with the GRU algorithm.
- **Efficiency and Robustness:** The GRU-based surrogate model outperformed other algorithms in terms of accuracy, computation time, and memory usage, showing a reduction of at least 29% in computation time and 50% in memory usage, highlighting the model’s efficiency and robustness.

## Figures and Tables

**Table 1:** Summary of process parameters.

Process Parameters (Unit)	Values
Laser Power (W)	600, 800, 1000
Scanning Speed (mm/s)	2, 4, 6
Hatching Space (%)	40, 50, 60
Laser Beam Size (mm)	2
Layer Thickness (mm)	0.5
Thermal Properties	Shown in Figure 4

**Table 2:** The twenty-seven-run design of experiment for multi-physics simulation.

Run	Laser Power (W)	Scanning Speed (mm/s)	Hatch Space (%)
1	600	2	60
2	600	2	50
3	600	2	40
4	600	4	60
5	600	4	50
6	600	4	40
7	600	6	60
8	600	6	50
9	600	6	40
10	800	2	60
11	800	2	50
12	800	2	40
13	800	4	60
14	800	4	50
15	800	4	40
16	800	6	60
17	800	6	50
18	800	6	40
19	1000	2	60
20	1000	2	50
21	1000	2	40
22	1000	4	60
23	1000	4	50
24	1000	4	40
25	1000	6	60
26	1000	6	50
27	1000	6	40

**Table 3:** Summary of training and testing data of surrogate models.

Model	Training Data	Testing Data	Training Size	Testing Size	Features	Labels
Melt Pool Peak Temperature	Run2-4, Run10-13, Run15-18, Run24-26	Run1, Run5, Run14, Run23, Run27	28,683	10,184	Time, Position X, Y, Z, Laser Power, Scanning Speed, Hatch Space	Melt Pool Peak Temperature
Melt Pool Dimension	Run2-4, Run10-13, Run15-18, Run24-26	Run1, Run5, Run14, Run23, Run27	20,182	7590	Time, Peak Temperature, Laser Power, Scanning Speed, Hatch Space	Melt Pool Length, Melt Pool Width, Melt Pool Depth

**Table 4:** Evaluation and comparative analysis: melt pool peak temperature model.

Algorithms	R-Square	RMSE	MAE	Computation Time (s)	Memory Usage (GB)
XGBoost	0.852	0.0550	0.0382	16.67	0.747
LSTM	0.979	0.0178	0.0126	238.60	2.41
Bi-LSTM	0.983	0.0153	0.0101	290.25	5.24
GRU	0.978	0.0179	0.0129	189.30	2.28

**Table 5:** Evaluation and comparative analysis: melt pool length model.

Algorithms	R-Square	RMSE	MAE	Computation Time (s)	Memory Usage (GB)
XGBoost	0.698	0.1031	0.0629	16.22	0.269
LSTM	0.888	0.0539	0.0412	76.23	1.37
Bi-LSTM	0.902	0.0501	0.0369	120.55	2.65
GRU	0.903	0.0503	0.0381	67.75	1.30

**Table 6:** Evaluation and comparative analysis: melt pool width model.

Algorithms	R-Square	RMSE	MAE	Computation Time (s)	Memory Usage (GB)
XGBoost	0.752	0.0963	0.0762	16.95	0.371
LSTM	0.946	0.0418	0.0313	86.26	1.37
Bi-LSTM	0.952	0.0399	0.0293	128.70	2.65
GRU	0.951	0.04	0.0291	76.73	1.30

**Table 7:** Evaluation and comparative analysis: melt pool depth model.

Algorithms	R-Square	RMSE	MAE	Computation Time (s)	Memory Usage (GB)
XGBoost	0.751	0.0892	0.0555	20.20	0.344
LSTM	0.871	0.0479	0.0360	97.69	1.44
Bi-LSTM	0.881	0.0476	0.0359	120.19	2.72
GRU	0.885	0.0420	0.0293	85.43	1.37

## Data Availability

The original contributions presented in the study are included in the article, further inquiries can be directed to the corresponding author.
